# Structured Monolithic Catalysts vs. Fixed Bed for the Oxidative Dehydrogenation of Propane

**DOI:** 10.3390/ma12060884

**Published:** 2019-03-16

**Authors:** Ilenia Rossetti, Elnaz Bahadori, Antonio Tripodi, Gianguido Ramis

**Affiliations:** 1 Chemical Plants and Industrial Chemistry Group, Dipartimento di Chimica, Università degli Studi di Milano, CNR-ISTM, INSTM Unit Milano Università, via C. Golgi 19, 20133 Milano, Italy; ilenia.rossetti@unimi.it (I.R.); antonio.tripodi@unimi.it (A.T.); 2 DICCA, Università degli Studi di Genova, and INSTM unit Genova, via all’Opera Pia 15A, 16100 Genoa, Italy; 713578@unige.it

**Keywords:** propene/propylene, oxidative dehydrogenation, olefins production, structured catalysts, structured reactors, V-based catalyst, primer, honeycomb monoliths

## Abstract

The deposition of V-based catalysts for the oxidative dehydrogenation of propane to propene on cordierite honeycomb monoliths was optimised as a strategy to decrease the contact time in a structured reactor with respect to a conventional fixed bed one. 10 wt% VO_x_ supported over SiO_2_ or Al_2_O_3_ were used as catalysts, deposed over the monolith using silica or alumina as primer, respectively. Both the alumina supported catalyst and the bohemite primer precursor were effectively deposed by dip-coating from stable powder suspensions, whereas insufficient adhesion was obtained when loading pre-synthesised SiO_2_ over the cordierite. A new method based on sol-gel production of SiO_2_ from tetraethylortosilicate (TEOS) over the monolith surface was set up. A correlation was derived for the prevision of the amount of silica deposed depending on the amount of TEOS. Both primer and catalyst loading were optimised as for uniformity and stability of the coating and resulted 0.5–1 wt % primer and 0.15 wt % of catalyst. Activity testing confirmed the strong improvement of propene productivity by increasing the time factor (i.e. Ncm^3^ of flowing reactant/min g_cat_), which ended in a one order of magnitude increase of productivity for the honeycomb-supported samples with respect to the fixed bed configuration.

## 1. Introduction

Low molecular weight olefins (C2–C4) are obtained mainly from steam cracking and catalytic cracking processes of natural gas and various oil fractions. From an application point of view, olefins are more interesting than their corresponding paraffins, since they are used as intermediates in various sectors of the basic chemical industry, especially in the polymers one [1,2]. Therefore, it is advantageous to transform compounds of low market price into products with higher added value. An important example is the dehydrogenation of isobutane to isobutene, whose demand on the market is constantly increasing, above all thanks to its application in the synthesis of methyltertbutylether (MTBE). The dehydrogenation of paraffins is an important way to obtain specific olefins for use in the polymer and intermediates industries.

Propene may be synthesized starting from propane through catalytic dehydrogenation, which however has some disadvantages: the reaction is thermodynamically limited by equilibrium and it is carried out at high temperature because of its endothermicity. Moreover, the formation of coke causes the deactivation of the catalyst, which must be frequently regenerated. The Oxidative Dehydrogenation (ODH) route is an interesting alternative, though not yet commercial, because it operates at a lower temperature and is an autothermal process. It also offers the advantage of removing coke through the oxygen co-fed. However, a serious drawback is represented by the co-production of CO and CO_2_ by complete combustion of propane and propene, where the latter is even more reactive than the former. This typically manifests in inverse proportionality between the conversion and selectivity trends. Furthermore, the coproduced water instead of hydrogen, lowers the overall value of the product mixture [3,4]. A trade-off should be searched between all these features to understand which is the best alternative.

The ODH reaction has been extensively studied by many research groups [5,6,7,8,9,10,11,12,13] and occurs through a redox mechanism, mediated by the catalyst:

C_3_H_8_ → C_3_H_6_ + 2H^+^ + 2 e^−^ (1)

2 H^+^ + ½ O_2_ + 2e^−^ → H_2_O (2)

for a total result of:

C_3_H_8_ + ½ O_2_ → C_3_H_6_ + H_2_O (propane ODH) (3)

This reaction obeys to a Mars-van Krevelen mechanism, where the catalyst active site, indicated as (M^n+^O)^(n−2)+^, is reduced by propane to M^(n−2)+^, which is in turn oxidised back by oxygen:

(M^n+^O)^(n−2)+^ + C_3_H_8_ → M^(n−2)+^ + C_3_H_6_ + H_2_O (4)

M^(n−2)+^ + ½ O_2_ → (M^n+^O)^(n−2)+^ (5)

Most of the catalysts studied for this type of reaction are transition metal oxides, thanks to their valuable redox properties than can arrange the two-electrons transfer required by this process, and among these, supported vanadium oxide proved the most active and selective one [14,15]. In this case, the reaction involves the reduction of the V^5+^ species to V^4+^ and then to V^3+^ in a reversible cycle, though other reaction paths indicate the need of adjacent sites for the reaction involving mainly the reduction to V^4+^ [16].

An important parameter is the amount of vanadium oxide on the support, which determines the nature of the active species, their dispersion and consequently influences the reaction mechanism [17,18,19,20,21,22,23,24,25]. It was demonstrated that the selectivity to propene was higher in the presence of isolated VO_x_ species. By increasing the vanadium loading, the isolated VO_x_ species tend to oligomerize, with the formation of V_2_O_5_ species: the higher the vanadium load, the greater the conversion, but the lower the propene selectivity, as vanadia favours the complete combustion to CO and CO_2_ [21,22,24,25,26,27].

The catalytic properties of vanadium for the ODH reaction are strongly influenced by the preparation method. In the recent past, we have investigated catalysts prepared by flame pyrolysis (FP), a peculiar method for the synthesis of solids that allows obtaining a nanometric powder, high surface area, and thermally stable materials [19,23,28,29]. A further advantage of this synthesis technique is represented by the possibility of synthesizing in a single step active phase and support in the desired proportions. In particular, we have compared VO_x_/SiO_2_ and VO_x_/Al_2_O_3_ achieving the desired better dispersion of the active species, with consequently higher selectivity to propene, than that achieved with traditional preparation methods, such as co-precipitation or impregnation over a preformed support. We also compared two different process alternatives, namely the co-feeding of propane and oxygen or the alternation of the reduction and oxidation steps, which has been differently called aerobic/anaerobic cycling, redox-decoupling, or chemical looping [19,23,30]. This latter option consisted of two separate reaction steps: i) the feeding of pure propane over the oxidised catalyst for a time corresponding roughly to the full reduction of the material; ii) the oxidation of the catalyst with pure oxygen to accomplish its regeneration. Much higher selectivity is ensured through the redox-decoupling procedure which, however, implies a more complex process design during scale up and continuous operation. 

On the other hand, the conversion/selectivity pattern also indicates that low contact time is another mean to improve the selectivity to propene when operating in continuous co-feed mode. Therefore, in this work, we have focused on the immobilisation of the catalyst over structured honeycomb monoliths as a strategy to decrease the amount of active material with respect to a conventional fixed bed configuration, so to conveniently decrease the contact time for a given propane + oxygen flowrate. In particular, we deepened here the preparation of the honeycomb coated monoliths comparing different preparation strategies, among which a sol-gel deposition of the precursor material, *vs*. dip-coating of preformed primers and catalysts. The selected catalysts were constituted by 10 wt% VO_x_/SiO_2_ (V10Si) and 10 wt% VO_x_/Al_2_O_3_ (V10Al), loaded on a 400 cpi cordierite honeycomb through the preliminary addition of a primer material to favour adhesion (samples HCSi and HCAl). The effect of loading conditions has been investigated and the catalytic activity of the optimised structured catalysts have been tested and compared with a fixed bed reactor configuration.

## 2. Results and Discussion

### 2.1. Optimisation of Catalyst Loading on Honeycombs

**Figure 1:**
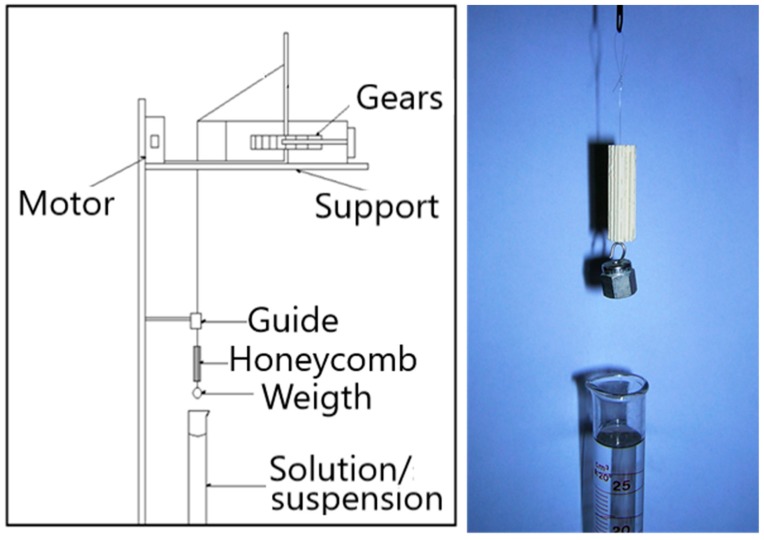
Schematic representation of the procedure for honeycomb loading.

An apparatus was set up in order to standardise the procedure for catalyst and primer loading on the preformed honeycombs [31,32]. The scheme of the apparatus is reported in Figure 1.

This apparatus allows to set and keep constant the dipping/extraction speed by selecting appropriate gears. Too high extraction speed led to an excessive and non-uniform deposition, with accumulation of the material in the lower part of the honeycomb. The optimal speed for immersing/extracting the honeycomb was 11.5 cm/min.

The preparation of the V10Si-coated honeycombs was set up using 2 or 4 cm long cylindrical samples of honeycomb (1 cm diameter), loaded with silica primer from different precursors, with various loading and deposition procedure, as sketched in the following Table 1 and in Table S1 (Electronic Supplementary Material). Final samples for activity testing were prepared from 5 cm long monoliths.

The resulting materials were at first visually inspected in order to identify visible defects such as exfoliation, cracks, or powder loss upon gentle shaking of the honeycombs. Unfortunately, adhesion was not quantified more rigorously since the standard adhesion test (scrape adhesion, cross cut, pull-off, etc.) are suitable for flat surfaces and extended coatings. Here the material is predominantly loaded inside the very small channels (400 cpi) of the honeycomb, which is a fragile material itself, and it is substantially impossible to access them without breaking the honeycomb, so affecting the stability of the coated layer. Even if it is not a very quantitative determination, shaking allowed immediately to collect possible detached power from the monolith and to select the candidates for further screening based on the absence of detached powder. Therefore, the apparently best samples were analysed through scanning electron microscopy (SEM).

The deposition of preformed silica powders led to unstable coatings, with poor adhesion (the suspension slides to the bottom of the honeycomb during calcination and silica detaches even during gentle manipulation of the honeycomb) and less uniform deposition. Furthermore, the procedure was difficult to standardise, since no apparent correlation emerged between the amount of silica in the suspension and the amount loaded. In every case the addition of HNO_3_ as peptising agent revealed effective to improve the adhesion and the uniformity of distribution.

The sol gel (SG) procedure for silica deposition from TEOS, in general, led to better primer homogeneity and dispersion. The Stöber process is an example of sol-gel polymerization of the Si alkoxide. TEOS is a suitable precursor for SG due to prompt reactivity with water through the attack of a hydroxyl moiety:

Si(OEt)_4_ + H_2_O → HO−Si(OEt)_3_ + R–OH (6)

Si(OEt)_4_ + 2 H_2_O → SiO_2_ + 4 R–OH (7)

**Figure 2:**
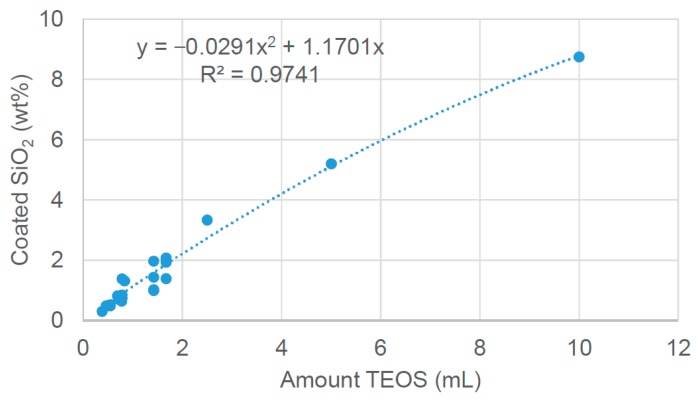
Correlation between the amount of TEOS used for deposition and the SiO_2_ loading. Fitting correlation: SiO_2_ (wt%) = −0.0291x^2^ + 1.1701x; x = mL of TEOS; R² = 0.9741.

Complete hydrolysis needs excess water or the presence of a hydrolysis catalyst such as an acid. The polymerisation of the precursor leads to the formation of siloxane bonds in a 3D network. This network was let grown on the cordierite surface and, most of all, the resulting material was stable to shaking and scratching after drying and calcination. The use of pure TEOS was not optimal, neither the addition of a very volatile solvent such as ethanol. The co-addition of water and sufficient hydrolysis time in controlled environment allowed a reproducible and stable deposition. Furthermore, the synthesis was reasonably controllable obtaining a good correlation between the volume of TEOS used and the SiO_2_ wt % loaded on the honeycomb (Figure 2).

**Figure 3:**
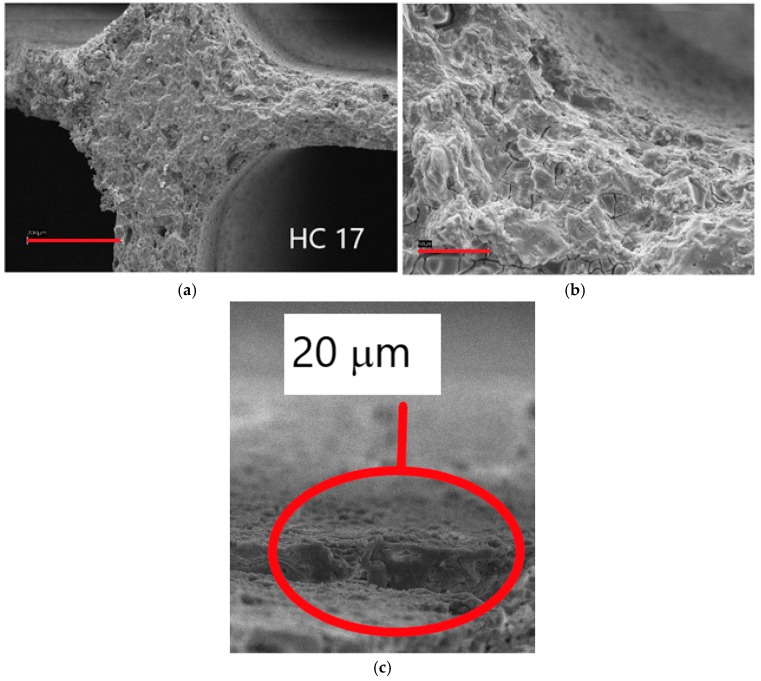
SEM micrographs of sample 17; marker size: (**a**) 300 μm; (**b**) 50 μm; (**c**) detail of the primer layer (20 μm average thickness).

One of the main parameters affecting the uniformity and stability of the coating was its loading. For instance, sample 17 from TEOS resulted in 3.3 wt % SiO_2_ deposed and its SEM micrographs are reported in Figure 3. Magnifying the apparently uniform primer layer, cracks appeared evident, suggesting to decrease the silica loading. The thickness of the primer layer was in average 20 μm, but it was not fully uniform on the surface, as evidenced in the same Figure 3.

**Figure 4:**
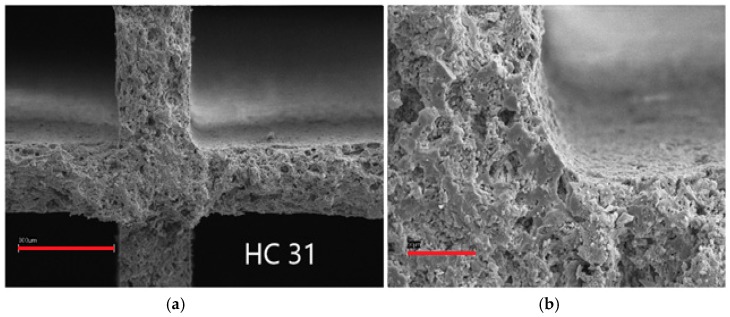
SEM micrographs of sample 31; marker size: (**a**) 300 μm; (**b**) 50 μm.

Sample 31 was characterised by 2.1 wt % SiO_2_ deposed, with significant improvement due to the absence of cracks, though still some accumulation of material was evident in the internal junction points, as evidenced in Figure 4, right side, where the red bars evidence different thickness of the layer, increasing in the internal curvature of the honeycomb.

**Figure 5:**
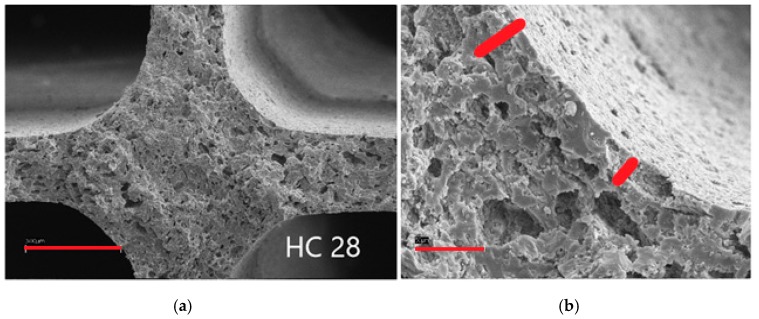
SEM micrographs of sample 28; marker size: (**a**) 300 μm; (**b**) 50 μm.

A further decrease of the SiO_2_ loading (1.03 wt %, HC 28, Figure 5) improved the uniformity of the primer layer and adhesion, without any cracks. Similar results were obtained by further decreasing the SiO_2_ loading to 0.46 wt % (HC 39), so that the optimal primer amount was set between 0.5 and 1 wt %. The thickness of the layer was *ca*. 10 μm, but difficult to assess on cut honeycombs due to damage imparted by the cutting medium.

As for the subsequent deposition of the catalyst by dip-coating, we opted for a two-step deposition, rather than the direct incorporation of primer and catalyst since the catalyst loading is very low and it should be effectively exposed on the surface without being embedded significantly into the primer layer, also to ensure the reproducibility of the synthesis and testing [31,32]. The same considerations drawn for the primer deposition hold also for the catalyst layer: an optimal loading should be searched to avoid cracks and fragility of the coating.

**Figure 6:**
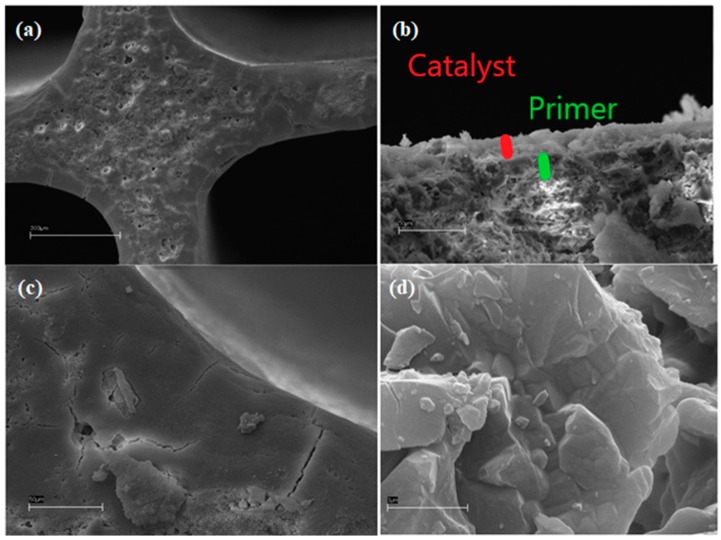
SEM micrographs of sample 22; marker size: (**a**) 300 μm; (**b**) 3 μm; (**c**) 50 μm; and (**d**) 3 μm.

For instance, the addition of 1.6 wt% of catalyst over 0.86 wt% SiO_2_ from TEOS (HC 22, Figure 6), was excessive, evidencing a very thick and dense catalyst layer with transversal cracks.

The two layers had thickness ca. 10–15 μm each, as shown in Figure 6b and showed perfect adhesion between them and with the support.

**Figure 7:**
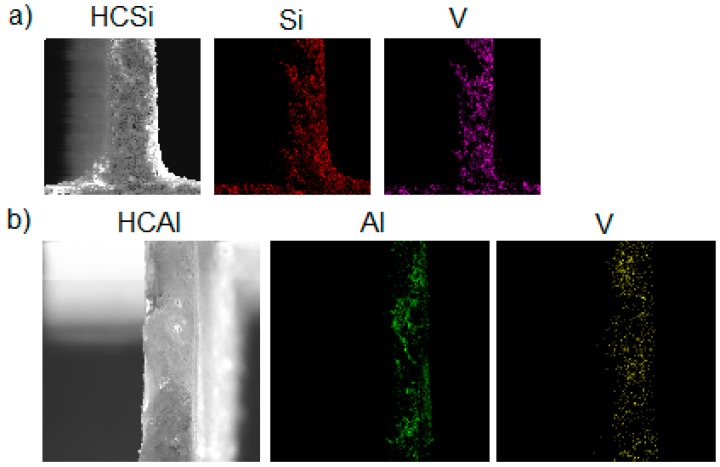
EDX maps of samples (**a**) HCSi and (**b**) HCAl.

The EDX maps of elements distribution is reported in Figure 7 for samples HCSi and HCAl, which show a uniform distribution of both Si and V on the surface of the support.

The addition of the primer layer also allowed to increase the surface area by one order of magnitude (Table 2). Indeed, the specific surface area of the bare cordierite honeycomb was 0.2 m^2^/g, while after loading the SiO_2_ primer (sample 28) it increased to 1.8 m^2^/g, with a contribution of micropores, determined from the t-plot, of ca. 0.7 m^2^/g and a total pore volume of 3.19 × 10^−4^ cm^3^/g. The addition of the V10Si catalyst, showing much higher surface area in powder form, did not affect significantly the surface area of the HCSi catalyst. The addition of the alumina primer increased the surface area to ca. 5 m^2^/g, slightly decreasing to ca. 3.5 m^2^/g after the addition of the catalyst layer.

### 2.2. Catalytic Activity

**Figure 8:**
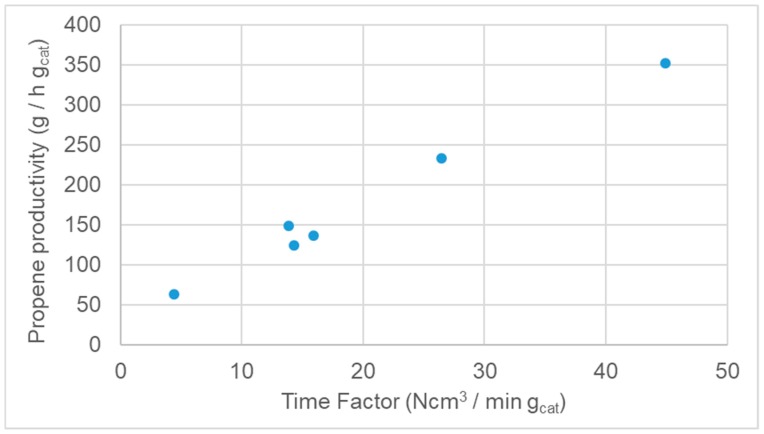
Propene productivity vs. time factor for catalyst V10Si (powder, fixed bed), T = 600 °C.

The catalysts were tested for the oxidative dehydrogenation of propane. At first, the effect of contact time in fixed bed configuration was tested over the most promising V10Si powder catalyst [19]. This sample was selected thanks to its good balance between conversion of propane and selectivity to propene. The effect of space velocity, i.e., the reciprocal of the contact time of the reactant over the active phase, is here expressed as a function of time factor (Ncm^3^/min g_cat_), as reported in Figure 8 for catalyst V10Si. The time factor has been selected as parameter because testing was carried out under constant reactants flow rate (to keep fixed the linear velocity and hence the fluid-dynamic regime) and decreasing the catalyst mass loaded in the fixed bed reactor. This directly allows a comparison with the structured catalyst, for which a catalyst volume to calculate the contact time would be misleading, given the different morphology of the samples. Time factors have been set by feeding the same flow of reactants (same fluid-dynamic conditions) and changing the catalyst mass. The time factor of 14 Ncm^3^/min g_cat_ was selected as a basis to achieve a significant conversion, though with unsatisfactory selectivity. Equal steps increasing and decreasing the parameter were done, so to obtain significant variation of the outcome (as in Figure 8), keeping both conversion and the selectivity variable and not flattening.

Increasing the time factor, induced an increase of propene productivity. This effect is mainly ascribed to a decrease of conversion, more effectively counterbalanced by higher selectivity to propene, with an overall increase of propene yield. In other words, increasing time factor means a reduction of the contact time, which allows higher gain of selectivity in spite of a loss of conversion.

**Figure 9:**
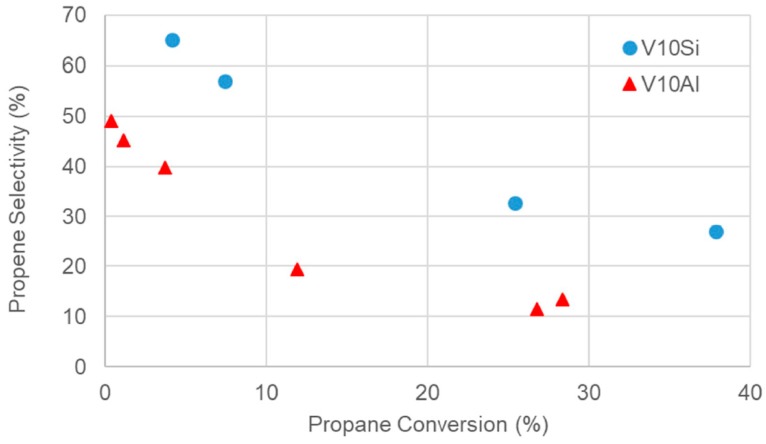
Propene selectivity vs. conversion for catalyst V10Si and V10Al (powder, fixed bed). Time factor = 14.3 Ncm^3^/min g_cat_. T = 450–600 °C for V10Si and 250–500 °C for V10Al, from left to right.

The decrease of selectivity at increasing conversion is exemplified in Figure 9 for the two catalysts V10Si and V10Al. In the same Figure 9 it is also evident the higher selectivity at isoconversion of the V10Si sample with respect to the V10Al. The reason for the higher selectivity of the silica supported VO_x_ was investigated through many different techniques. In particular, V^4+^ ions were analysed by Electron Paramagnetic Resonance (EPR) [22,33], showing the typical pattern of isolated, tetragonally distorted, paramagnetic complexes of V^4+^ forming a monolayer on the surface with a strong out-of-plane V^4+^–O bond for the V10Si catalyst. With a comparative sample prepared by impregnation of the V precursor rather than by direct flame pyrolysis synthesis this bond was a bit weaker and ferromagnetic domains of clustered V ions were present, indicating a higher dispersion of V for the FP prepared sample. Much weaker V^4+^–O bond was detected for the V10Al sample, which means on one hand a higher oxygen availability, thus higher conversion (at lower temperature, as evidenced in Figure 9), but lower selectivity. Additionally, for this formulation the impregnation method was less effective to impart high V dispersion, which is another key factor to enhance selectivity. These conclusions on dispersion were also confirmed by micro-Raman spectroscopy [19,23] and X-ray absorption spectroscopy (XAS) [21]. The higher V dispersion attainable with the FP technique may be ascribed to the flash calcination at high temperature. This quenches the system as a metastable structure, where V remains finely incorporated into the support matrix as in a mixed oxide. Analogies in V local structure between the V10Si and V10Al with the mixed AlVO_4_ compound were assessed, though in V10Al come V_2_O_5_ contribution also appeared, contributing to the lower selectivity. The details on reaction mechanism and on kinetics for this reaction over Mg-V-Sb oxide has been detailed by Grasselli et al. [34], evidencing as rate determining the interaction of the adsorbed propane with activated oxygen species over the catalyst surface.

The effect of the activation temperature was also tested (Table 3) evidencing an increase of propene productivity with this parameter irrespectively of the time factor. 

The productivity boosted by one order of magnitude for the honeycomb-supported catalysts. The strategy of dispersing the active phase in very small amount to improve propene yield proved effective. This was especially true for the V10Al, for which the enhanced time factor, i.e., lower contact time, allowed to decrease effectively the conversion with positive consequence on propene selectivity. This sample was indeed less selective than the SiO2 supported one, as specified above, due to lower VOx dispersion. Therefore, the effect of selectivity improvement at lower conversion was much more evident for V10Al. The effect on productivity can be also quantified by a parameter, here newly introduced and called enhancement factor (Table 3), defined as:

$$\mathrm{Enhancement}\;\mathrm{Factor}=\frac{\left(\frac{\mathrm{Productivity}\;\mathrm{HC}-\mathrm{supported}\;\mathrm{catalyst}}{\mathrm{Productivity}\;\mathrm{unsupported}\;\mathrm{catalyst}}\right)}{\left(\frac{\mathrm{Time}\;\mathrm{Factor}\;\mathrm{HC}-\mathrm{supported}\;\mathrm{catalyst}}{\mathrm{Time}\;\mathrm{Factor}\;\mathrm{unsupported}\;\mathrm{catalyst}}\right)}$$

Enhancement factors equal to 1 when the increase of productivity (higher yield) is due only to the increase of time factor. By contrast, a value lower than 1, as for HCSi and HCAl, indicates that the system is tested at so high time factor to be in a quasi-plateau zone of the curve exemplified in Figure 8, when further increasing the time factor. Indeed, above a given value of time factor, no further enhancement of selectivity is possible while decreasing the conversion, flattening out the productivity. This was especially true for V10-Si, for which the enhancement factor was ca. 0.2, whereas for V10Al it was ca. 0.5. This testifies on one hand the extremely beneficial effect of honeycomb supporting, especially in the case of the less selective catalysts. On the other hand this factor can be used to optimise catalyst loading to a value after which there is no additional advantage of enhanced productivity.

Overall, suitable supporting over monolithic honeycomb and improved propene productivity can be achieved by deposing on the support ca. 0.5–1 wt % primer and ca. 0.15 wt % of catalyst. At last, we have tested our catalysts at least for 10 h-on-stream, at increasing temperature, with steps of 2 h for each temperature, with stable performance under isothermal conditions, as a first confirmation that no fast deactivation is occurring (e.g., coking at low temperature).

The conversion/selectivity data reported in Figure 9 are comparable with those reported by Viparelli et al. [35], who used 6% Nb or 1% V + 6% Nb over TiO_2_. A bulk V_2_O_5_ catalyst has been tested by Garcìa et al. [36], obtaining as expected very limited selectivity to propene (max. ca. 40% at 300 °C) even with propane conversion as low as 5%; ca. 50% conversion at 550 °C but with only 1% selectivity to propene has been reported by Al-Ghamdi et al. [37], while 53% selectivity was obtained by Solsona et al. with 21% conversion of propane over a V-Si material (ITQ-6) [27].

## 3. Materials and Methods

### 3.1. Catalyst Preparation

All the catalysts taken into consideration in this work were prepared by flame pyrolysis (FP) as better detailed elsewhere [19,23,28,29] and were constituted by VO_x_ supported over SiO_2_ or Al_2_O_3_. Catalyst codes are here presented as V10Si or V10Al, when used in powder form, where the number indicates a 10 wt% VO_x_ loading, expressed as V_2_O_5_. 

Briefly, the catalyst precursors were V oxy-acetylacetonate (Merck, Kenilworth, NJ, USA, 98%), tetraethylortosilicate (TEOS, Sigma-Aldrich, St. Louis, MO, USA, purity 98%) and Al(NO_3_)_3_·9H_2_O (Sigma-Aldrich, St. Louis, MO, USA, purity 98%).

The precursors in the desired amount were dissolved in an organic solvent, composed of 1:1 (*V*/*V*) ethanol (Fluka, Bucharest, Romania, 99.8%) + octanol (Fluka 99.0%).

For the FP synthesis, the precursor solution, with a 0.1 M concentration, was fed to a home designed burner with a flowrate of 4.4 mL/min and co-fed with 5 L/min of oxygen. The pressure drop at the nozzle was set as 0.4 bar.

### 3.2. Catalyst Deposition on Honeycombs

A commercial cordierite (Mg_2_Si_5_Al_4_O_18_) support was used to load the catalyst powder, extruded in the form of honeycomb monoliths, with channels of constant dimensions arranged longitudinally to the gas flow. The honeycombs had a height of 5 cm and a diameter of 1 cm and were prepared by drilling a commercial cordierite honeycomb block. Since the honeycomb has a low surface area, it was coated with a primer layer, which supported the catalyst and increased the surface area, favouring the adhesion. The deposition of the FP catalyst powder on the honeycomb was carried out by dip-coating, immersing the honeycomb in a stable suspension of the catalyst powder, with a constant immersion and extraction speed, in order to have a homogeneous powder deposition on the whole support. The rates were kept constant thanks to a stepper motor that maintains constant the speed of rotation of shaft on which a nylon wire (carrying the honeycomb) is wound. The motor is connected to the wire by a series of gears, whose diameter can be changed, thus varying the dipping/extraction speed.

Before loading the catalyst, a primer was deposed on the cordierite honeycomb. Two methods were compared, namely a sol-gel procedure to depose the oxide primer over the support, or dip-coating of a preformed oxide. The main operating parameters, such as the primer precursor, the concentration of the solution (for sol-gel) or suspension (for dip-coating), the dispersing medium, the presence or absence of a peptizing agent were investigated and are discussed in paragraph 2. 

When needed, the preparation of stable suspensions of preformed powder (catalyst or primer) was accomplished by prolonged wet grinding in a small ball mill for 16 h. A 50 mL polypropylene closed flask with 4 zirconia balls was mounted on a twirl. The zirconia balls with their continuous bumping and mixing allowed to grind the powder inside the container, breaking up the agglomerates and stabilising the suspension. 

For the V10Si catalyst, SiO_2_ was used as primer, deposed either by sol-gel method using TEOS as silica precursor, or by using SiO_2_ powder prepared by FP or Cab-o-sil^®^ (amorphous fumed silica, Sigma-Aldrich, purity 99.8%) for the dip-coating procedure. For the V10Al sample the primer was deposed by dip-coating with boehmite (Disperal^®^, Sasol, Johannesburg, South Africa, purity 99.9%). After each deposition the honeycomb was dried in an oven at 90–110 °C for ca. 30 min and then calcined at 530 °C for 30 min in a muffle (static oven). During drying and calcination the honeycomb was suspended in a vertical position if not otherwise specified in Table S1.

### 3.3. Catalysts Characterisation

For a detailed characterisation of the powder catalysts the reader is referred to previously published work [19,20,21,22,23,24,25].

The honeycomb supported catalysts were characterised by scanning electron microscopy (SEM) by means of a LEICA LEO 1430 instrument.

The specific surface area of the materials (SSA) was determined by N_2_ adsorption/desorption at −196 °C using a Micromeritics ASAP2010 apparatus (Norcross, GA, USA), equipped with a specifically designed sample tray to hold the honeycombs and the data were elaborated through the BET algorithm. All the samples were previously outgassed overnight at 300 °C.

### 3.4. Catalytic Activity Testing

Activity tests were carried out in a quartz tubular reactor, 370 mm long and with an internal diameter of 7 mm, for granular catalysts in fixed bed configuration. A steel tubular reactor was used for tests on catalysts deposited on honeycomb, 400 mm long and with an internal diameter of 10.5 mm. A tubular oven allowed to control the temperature of the reactors, connected to a Temperature Indicator Controller (TIC). The reactors were placed inside the oven enclosed between two stainless steel fittings (Aisi 316). Mass flowmeters were used to set the desired inlet flow.

The outlet gas composition was analysed by an in-line gas chromatograph (GC, HP 5890 HWD).The activity tests in fixed bed configuration were carried out on the catalyst powder formed by pressing, grinding and sieving in 0.15–0.25 mm granules. The catalyst was diluted 1:5 (*w*/*w*) with quartz granules of the same size. The mixture was kept in the centre of the reactor by two layers of quartz wool and the remaining void space of the reactor on top and below the catalyst bed was filled with quartz granules of larger size. The catalytic bed was ca. 5 cm long, containing ca. 1.5 grams of the catalyst-quartz mixture.

As for the activity tests carried out on honeycomb, the steel reactor was loaded similarly to the quartz one, but with the honeycomb positioned at the centre of the reactor instead of the powder.Catalyst activation was carried out with a 20 Ncm^3^/min air flow, heating by 10 °C/min up to 600 °C, kept for 1 h. Higher temperature was also tested as specified in Table 2. 

The activity test was carried out by feeding 4 mol% propane (SAPIO, Monza, Italy, purity 99.5%), 4 mol% oxygen (SAPIO, purity 99.90%), 89 mol% helium (SAPIO, purity 99.996%) as inert gas and 3 mol% nitrogen (SAPIO, purity 99.9990%) as an internal reference standard for gas-chromatographic analysis. The activity tests were carried out from 200 °C up to 600 °C and carrying out three GC analyses of the effluent gas every 50 °C.

## 4. Conclusions

The oxidative dehydrogenation of propane to propene was carried out on different V-based catalysts, either supported over silica or alumina. The decrease of contact time improved propene productivity in fixed bed configuration and this suggested to support the active phase over a cordierite honeycomb to improve its dispersion and to lower by orders of magnitude the catalyst loading, so increasing the time factor.

The deposition of the catalyst over the structured support required the preliminary addition of a primer, selected as SiO_2_ or Al_2_O_3_ depending on the support of the active phase. The primer and catalyst deposition methods were optimised, especially for SiO_2_, since the attempts of deposition of preliminary prepared SiO_2_ (either home-synthesised by FP or commercial Cab-o-sil^®^) resulted in unstable coating with poor adhesion, at difference with Al_2_O_3_, effectively coated by dip-coating of Disperal^®^ bohemite. 

Therefore, a different strategy was set up, developing a sol-gel procedure for the direct deposition of silica on the honeycomb starting from TEOS as precursor. The primer deposition was optimised also searching for a correlation between the amount of TEOS and the silica loading. The amount of primer and of active phase were optimised to achieve satisfactory adhesion and uniform deposition. This was achieved with ca. 0.5–1 wt% primer and ca. 0.15 wt% of catalyst.

Activity testing confirmed the importance of the decrease of the catalyst amount, through its uniform dispersion over the structured support. Indeed, the productivity increased for the honeycomb supported catalysts by one order of magnitude. The enhancement factor of propene productivity was more sensitive for the less selective V10Al catalyst.

## Figures and Tables

**Table 1:** Details of the preparation of the suspension of the V10Si catalyst powder for the dip-coating. a: The suspension was diluted 1:2 (*V*/*V*) after ball milling. The V10Al sample was loaded as for preparation “L”.

Suspension	Catalyst Amount (g)	H_2_O Volume (mL)	HNO_3_ (mL)	Ball-Milling Time (h)
A	0.4064	25	0.15	12
B	0.4032	25 ^a^	0.15	12
C	0.1487	25	0.15	24
D	0.1961	25	0.15	24
E	0.4019	25	0.15	48
F	0.3382	25	0.15	24
G	0.1010	25	0.15	24
H	0.2010	25	0.15	24
I	0.0633	25	0.15	24
L	0.0688	25	0.25	24

**Table 2:** BET specific surface area (SSA) and porosity data for selected samples obtained from N_2_ adsorption/desorption at 77 K. Micropore area was estimated from t-plot.

Sample	SSA (m^2^/g)	Micropore Area (m^2^/g)	Pore Volume (cm^3^/g)
V10Si (powder)	37	13	0.09
V10Al (powder)	20	14	0.07
Honeycomb	0.2	-	-
Sample 28 (only primer)	1.8	0.7	3.19 × 10^−4^
Sample 50 (only primer)	4.7	2.5	0.011
HCSi (sample 49)	2.2	1.0	0.008
HCAl (sample 50)	3.5	2.1	0.011

**Table 3:** Propene productivity for different catalysts. Samples labelled HC indicate honeycomb supported ones, else powdered samples in fixed bed reactor. Samples numbers as in Table S1.

Catalyst	Activation Temperature (°C)	Time Factor (Ncm^3^/min g_cat_)	Productivity (g/h g_cat_)	Enhancement Factor
V10Si	600	14.3	124	-
V10Si	800	14.3	164	-
V10Si	600	26.4	233	-
V10Si	700	21.2	251	-
V10Al	600	21.9	98	-
HCSi (sample 49)	600	1079.9	1752	0.18
HCAl (sample 50)	600	1030.5	2121	0.46

## Supplementary Materials

The following are available online at https://www.mdpi.com/1996-1944/12/6/884/s1, Table S1: Details of the deposition of the primer on the honeycombs. a: DC = Dip-coating from primer powder suspension, SG = Sol-gel deposition of the primer; b: bm = ball milling; c: catalyst suspension as for Table 1 for the preparation of honeycomb/primer/catalyst samples.
