# Supplementary material for: Structured Monolithic Catalysts vs. Fixed Bed for the Oxidative Dehydrogenation of Propane
Source: Materials (Basel). 2019 Mar 16;12(6):884. doi: 10.3390/ma12060884 (PMC6471166; doi:10.3390/ma12060884)
Supplement: Supplementary file 1 [file materials-12-00884-s001.pdf]

materials

Article

Structured Monolithic Catalysts vs. Fixed Bed for the Oxidative Dehydrogenation of Propane

Ilenia Rossetti [1], Elnaz Bahadori [2], Antonio Tripodi [1] and Gianguido Ramis [2,*]

1 Chemical Plants and Industrial Chemistry Group, Dipartimento di Chimica, Università degli Studi di Milano, CNR-ISTM, INSTM Unit Milano Università, via C. Golgi 19, 20133 Milano, Italy; ilenia.rossetti@unimi.it (I.R.); antonio.tripodi@unimi.it (A.T.)

2 DICCA, Università degli Studi di Genova, and INSTM unit Genova, via all'Opera Pia 15A, 16100 Genoa, Italy; 713578@unige.it

* Correspondence: gianguidoramis@unige.it

Table S1. Details of the deposition of the primer on the honeycombs. a: DC = Dip-coating from primer powder suspension, SG = Sol-gel deposition of the primer; b: bm = ball milling; c: catalyst suspension as for Table 1 for the preparation of honeycomb/primer/catalyst samples.

Sample	Primer (method) [a]	Primer preparation [b]	Calcination	Primer Loading (wt%)	Catalyst Loading (wt%)	Comments	Catalyst suspension [c]
1	SiO_2 (DC)	0.7999 g SiO_2 + 30 mL H_2O + 0.1 mL HNO_3, bm 1 h	1 h in static oven 530 °C, horizontal	0.71	-	The prime does not adhere, SiO_2 powder loss.	-
2	TEOS (SG)	Pure	1 h in static oven 530 °C, horizontal	0.39 (1st) 1.04 (2nd)	-	Too rapid decomposition, not uniform deposition	-
3	SiO_2 (DC)	0.7999 g SiO_2 + 30 mL H_2O + 0.15 mL HNO_3, bm 12 h	1 h in static oven 530 °C, horizontal	2.10	1.04	The prime does not adhere, SiO_2 powder loss. Not uniform	A
4	TEOS (SG)	Pure	10 min 100 °C, 1 h in static oven 530 °C, horizontal	0.28	-	Silica accumulated in many points	
5	CAB-O-SIL (DC)	0.8002 g + 30 mL H_2O + 0.15 mL HNO_3, bm 12 h	1 h in static oven 530 °C, horizontal	2.54	-	As case 6	
6	CAB-O-SIL (DC)	0.3949 g + 30 mL H_2O + 0.1 mL HNO_3, bm 1h	1 h in static oven 530 °C, horizontal	0.84	0.68	Unstable primer suspension; the catalyst layer does not adhere.	A
7	CAB-O-SIL (DC)	0.3949 g + 30 mL H_2O + 0.05 mL HNO_3, bm 1 h	1 h in static oven 530 °C, horizontal	1.28	-	Unstable primer suspension.	
8	SiO_2 (DC)	0.4126 g + 30 mL H_2O + 0.15 mL HNO_3, bm 1 h	1 h in static oven 530 °C, horizontal	-	-	Unstable primer suspension.	
9	TEOS (SG)	pure	Tubulat oven 30′ at 170 °C (10 °C/min, in air) + 1 h in static oven 530 °C, vertical	2.64	-	Too thick layer and not uniform.	
10	SiO_2 (DC)	0.4126 g + 30 mL H_2O + 0.15 mL HNO_3, bm 5 h	1 h in static oven 530 °C, vertical	-	-	The adhesion is insufficinet. Impossible the vertical position for calcination (powder loss in the bottom during calcination)	
11	TEOS (SG)	TEOS:Ethanol = 1:2 (v/v)	As for 9	0.03	-	Too fast evaporation of the solvent	
12	TEOS (SG)	TEOS:Ethanol = 1:2 (v/v)	0.5 h in static oven, 100 °C, vertical	-		Too fast evaporation of the solvent	

13	TEOS (SG)	TEOS:1-propanol = 1:2 (v/v)	0.5 h in static oven, 100 °C, vertical	-	-	Too fast evaporation of the solvent	
14	SiO_2 (DC)	0.4126 g + 30 mL H_2O + 0.15 mL HNO_3, bm 12 h	1 h in static oven 530 °C, horizontal	2.08	-	Not uniform	
15	TEOS (SG)	7.5 mL TEOS + 7.5 mL H_2O + 7.5 mL Ethanol + 0.25 mL HNO_3	0.5 h in static oven at 100 °C, then 0.5 h in static oven 530 °C, vertical	8.75	-	Stable layer	
16	TEOS (SG)	5 mL TEOS + 10 mL H_2O + 10 mL Ethanol + 0.25 mL HNO_3	0.5 h in static oven at 100 °C, then 0.5 h in static oven 530 °C, vertical	5.21	-	Stable layer	
17	TEOS (SG)	2.5 mL TEOS + 10 mL H_2O + 10 mL Ethanol + 0.25 mL HNO_3	0.5 h in static oven at 100 °C, then 0.5 h in static oven 530 °C, vertical	3.34	-	Stable layer	
18	TEOS (SG)	1 mL TEOS + 12 mL H_2O + 12 mL Ethanol + 0.25 mL HNO_3	0.5 h in static oven at 100 °C, then 0.5 h in static oven 530 °C, vertical	1.32	-	Stable layer	
19	TEOS (SG)	1 mL TEOS + 13 mL H_2O + 13 mL Ethanol + 0.25 mL HNO_3	0.5 h in static oven at 100 °C, then 0.5 h in static oven 530 °C, vertical	1.39	-	Stable layer	
20	TEOS (SG)	1 mL TEOS + 14 mL H_2O + 14 mL Ethanol + 0.25 mL HNO_3	0.5 h in static oven at 100 °C, then 0.5 h in static oven 530 °C, vertical	0.80	-	Stable layer	
21	SiO_2 (DC)	0.2022 g SiO_2 + 30 mL H_2O + 0.25 HNO_3	1 h in static oven 530 °C, horizontal	0.43	-	Stable layer.	
22	TEOS (SG)	1 mL TEOS + 13 mL H_2O + 13 mL Ethanol + 0.25 mL HNO_3	0.5 h in static oven at 100 °C, then 0.5 h in static oven 530 °C, vertical	0.86	1.57	Stable layer.	A
23	TEOS (SG)	1.7 mL TEOS + 12 mL H_2O + 12 mL EtOH + 0.25 mL HNO_3	0.5 h in static oven at 100 °C, then 0.5 h in static oven 530 °C, vertical	1.98	5.11	Stable primer layer, excess of catalyst	A
24	TEOS (SG)	Hc 4 cm; 1 mL TEOS + 12 mL H_2O + 12 mL Ethanol + 0.25 mL HNO_3	0.5 h in static oven at 100 °C, then 0.5 h in static oven 530 °C, vertical	0.82	1.22	The amount deposed depends on HC lenght.	A

25	TEOS (SG)	1.7 mL TEOS + 12 mL H_2O + 12 mL Ethanol + 0.25 mL HNO_3	0.5 h in static oven at 100 °C, then 0.5 h in static oven 530 °C, vertical	1.44	-	Stable layer.	
26	TEOS (SG)	1 mL TEOS + 13 mL H_2O + 13 mL Ethanol + 0.25 mL HNO_3	0.5 h in static oven at 100 °C, then 0.5 h in static oven 530 °C, vertical	0.75	1.03	Stable layers.	B
27	TEOS (SG)	0.9 mL TEOS + 13 mL H_2O + 13 mL Ethanol + 0.25 mL HNO_3	0.5 h in static oven at 100 °C, then 0.5 h in static oven 530 °C, vertical	0.83	-	Stable layer.	
28	TEOS (SG)	1.7 mL TEOS + 12 mL H_2O + 12 mL Ethanol + 0.25 mL HNO_3	0.5 h in static oven at 100 °C, then 0.5 h in static oven 530 °C, vertical	1.03	-	Stable layer.	
29	TEOS (SG)	1.7 mL TEOS + 12 mL H_2O + 12 mL Ethanol + 0.25 mL HNO_3	0.5 h in static oven at 100 °C, then 0.5 h in static oven 530 °C, vertical	1	-	Stable layer.	
30	TEOS (SG)	Hc 5 cm; 1.5 mL TEOS + 12 mL H_2O + 12 mL EtOH + 0.25 mL HNO_3	0.5 h in static oven at 100 °C, then 0.5 h in static oven 530 °C, vertical	0.73	0.36	Stable layers.	D
31	TEOS (SG)	2 mL TEOS + 12 mL H_2O + 12 mL Ethanol + 0.25 mL HNO_3	0.5 h in static oven at 100 °C, then 0.5 h in static oven 530 °C, vertical	2.08	-	Stable layer.	
32	TEOS (SG)	2 mL TEOS + 12 mL H_2O + 12 mL Ethanol + 0.25 mL HNO_3	0.5 h in static oven at 100 °C, then 0.5 h in static oven 530 °C, vertical	1.94	0.56	Stable layer, unstable suspension of catalyst.	C
33	TEOS (SG)	2 mL TEOS + 12 mL H_2O + 12 mL Ethanol + 0.25 mL HNO_3	0.5 h in static oven at 100 °C, then 0.5 h in static oven 530 °C, vertical	1.4	0.73	Stable layers.	D
34	TEOS (SG)	Hc 5 cm; 1 mL TEOS + 12 mL H_2O + 12 mL EtOH + 0.25 mL HNO_3	0.5 h in static oven at 100 °C, then 0.5 h in static oven 530 °C, vertical	0.95	-	Stable layers.	E
35	TEOS (SG)	1 mL TEOS + 13 mL H_2O + 13 mL Ethanol + 0.25 mL HNO_3	0.5 h in static oven at 100 °C, then 0.5 h in static oven 530 °C, vertical	0.65	-	Stable layer.	

36	TEOS (SG)	1 mL TEOS + 13 mL H_2O + 13 mL Ethanol + 0.25 mL HNO_3	0.5 h in static oven at 100 °C, then 0.5 h in static oven 530 °C, vertical	0.71	1.02	Stable layers.	F
37	TEOS (SG)	1 mL TEOS + 14 mL H_2O + 14 mL Ethanol + 0.25 mL HNO_3	0.5 h in static oven at 100 °C, then 0.5 h in static oven 530 °C, vertical	0.92	-	Stable layer. Optimal result.	
38	TEOS (SG)	1 mL TEOS + 14 mL H_2O + 14 mL Ethanol + 0.25 mL HNO_3	0.5 h in static oven at 100 °C, then 0.5 h in static oven 530 °C, vertical	0.71	0.37	Stable layers.	G
39	TEOS (SG)	1 mL TEOS + 15.5 mL H_2O + 15.5 mL Ethanol + 0.25 mL HNO_3	0.5 h in static oven at 100 °C, then 0.5 h in static oven 530 °C, vertical	0.46	-	Stable layer. Optimal result.	
40	TEOS (SG)	0.5 mL TEOS + 13 mL H_2O + 13 mL Ethanol + 0.25 mL HNO_3	0.5 h in static oven at 100 °C, then 0.5 h in static oven 530 °C, vertical	0.31	-	Stable layer.	
41	TEOS (SG)	0.7 mL TEOS + 13 mL H_2O + 13 mL Ethanol + 0.25 mL HNO_3	0.5 h in static oven at 100 °C, then 0.5 h in static oven 530 °C, vertical	0.53	-	Stable layer.	
42	TEOS (SG)	0.5 mL TEOS + 15 mL H_2O + 15 mL Ethanol + 0.25 mL HNO_3	0.5 h in static oven at 100 °C, then 0.5 h in static oven 530 °C, vertical	0.39	0.37	Stable layers.	G
43	TEOS (SG)	Hc 4 cm<br>1–0.6 mL TEOS + 12 mL H_2O + 12 mL Ethanol + 0.25 mL HNO_3<br>2–same suspension	Both for 1 and 2:0.5 h in static oven at 100 °C, then 0.5 h in static oven 530 °C, vertical	0.29 (1st)<br>0.72 (2nd)	0.85	Stable layers. Double TEOS deposition.	H
44	TEOS (SG)	Hc 4 cm; 0.8 mL TEOS + 13 mL H_2O + 13 mL Ethanol + 0.25 mL HNO_3	0.5 h in static oven at 100 °C, then 0.5 h in static oven 530 °C, vertical	0.48	-	Stable layer.	
45	TEOS (SG)	Hc 4 cm; 0.8 mL TEOS + 13 mL H_2O + 13 mL Ethanol + 0.25 mL HNO_3	0.5 h in static oven at 100 °C, then 0.5 h in static oven 530 °C, vertical	0.39	0.51	Stable layers.	H

46	TEOS (SG)	0.6 mL TEOS + 13 mL H_2O + 13 mL Ethanol + 0.25 mL HNO_3	0.5 h in static oven at 100 °C, then 0.5 h in static oven 530 °C, vertical	0.50	0.24	Stable layers.	I
47	TEOS (SG)	0.6 mL TEOS + 13 mL H_2O + 13 mL Ethanol + 0.25 mL HNO_3	0.5 h in static oven at 100 °C, then 0.5 h in static oven 530 °C, vertical	0.54	(0.24) 0.59	Stable layers. Double deposition of active phase.	I
48	TEOS (SG)	0.7 mL TEOS + 13 mL H_2O + 13 mL Ethanol + 0.25 mL HNO_3	0.5 h in static oven at 100 °C, then 0.5 h in static oven 530 °C, vertical	0.49	-	Stable layer.	
49	TEOS	HC 5 cm; 1 mL TEOS + 15 mL H_2O + 15 mL Ethanol + 0.25 mL HNO_3	0.5 h in static oven at 100 °C, then 0.5 h in static oven 530 °C, vertical	0.46	0.16	Stable layers.	L
50	Disperal (Condea Chemie) Boehmite, 99.9%	HC 5 cm; 0.2075 g Dispersal in 22.5 mL H_2O + 0.4 mL HNO_3	0.5 h in static oven at 100 °C, then 0.5 h in static oven 530 °C, vertical	0.36	0.17	Stable layers.	L

© 2019 by the authors. Submitted for possible open access publication under the terms and conditions of the Creative Commons Attribution (CC BY) license (http://creativecommons.org/licenses/by/4.0/).
